# T790M mutation positive squamous cell carcinoma transformation from EGFR-mutated lung adenocarcinoma after low dose erlotinib: A case report and literature review

**DOI:** 10.1097/MD.0000000000029682

**Published:** 2022-08-12

**Authors:** Yusaku Kusaba, Yuichiro Takeda, Sakurako Abe, Akinari Tsukada, Go Naka

**Affiliations:** Department of Respiratory Medicine, National Center for Global Health and Medicine, Tokyo, Japan.

**Keywords:** case report, histologic transformation, nonsmall cell lung cancer, Osimertinib, tyrosine kinase inhibitor

## Abstract

**Rationale:**

Epidermal growth factor receptor (EGFR) tyrosine kinase inhibitors (TKIs) are widely used for the treatment of EGFR mutation positive advanced nonsmall cell lung cancer (NSCLC); however, acquired resistance is known to develop during these treatments. Among these mechanisms, histological transformation is seldom encountered. Although platinum based chemotherapy has been reported to be effective in the treatment of patients with small cell lung cancer transformation, there is a lack of information on the treatment of patients with squamous cell carcinoma (SQ) transformation.

**Patient Concerns and Diagnosis:**

An 80-year-old nonsmoking woman was referred to our hospital because of an abnormal shadow on her chest radiograph. Diagnostic bronchoscopy was performed and pathological examination revealed adenocarcinoma. Mutation analysis of the EGFR gene revealed deletion of E746-A750 in exon 19. She refused both surgical treatment and radiation therapy, and preferred periodic radiologic follow-up. Unfortunately, approximately a year and a half after the initial diagnosis, the primary lesion enlarged, and many pleural nodules were newly detected (clinically T4N2M1a, stage IVA).

**Interventions and Outcomes:**

Based on EGFR mutation analysis, a reduced dose of daily erlotinib was prescribed, which achieved a partial response and 34 months of progression-free survival (PFS). A repeated biopsy with an endobronchial cryoprobe was performed on the enlarged primary lesion. Pathological examination revealed SQ harboring an identical EGFR mutation with a secondary EGFR T790M mutation. Osimertinib 80 mg once a day was started as second line therapy, which resulted in 8 months of PFS and 15 months of survival.

**Lesson:**

The literature review and our report suggest that osimertinib is a promising treatment for NSCLC regardless of histology if T790M is present as an acquired mutation.

## 1. Introduction

Epidermal growth factor receptor tyrosine kinase inhibitors (EGFR TKIs) are widely used in EGFR mutation positive advanced nonsmall cell lung cancer (NSCLC). Tumors are known to acquire resistance to EGFR TKIs as first line treatment. In such circumstances, tumor rebiopsy is recommended as a second line therapy, especially after the failure of first or second generation EGFR TKIs.^[1]^ Two main types of resistance mechanisms to EGFR mutated NSCLC are “on target” and “off target” resistance.^[2]^ The former is the secondary alteration in the target oncogene, including either a second site mutation that promotes TKI resistance or the amplification or loss of the targeted oncogene. The EGFR T790M mutation is found in >50% of patients with acquired resistance to early generation EGFR TKIs,^[3,4]^ which occurs at a conserved “gatekeeper” threonine residue within the ATP binding pocket. EGFR T790M mutation is sensitive to osimertinib as a second line therapy.^[5]^ The latter is a tumor cell alteration occurring in proteins other than the targeted oncoprotein, which includes downstream signaling pathways and parallel bypass signaling pathways.

Histologic transformation is a type of resistance mechanism that is not a feature of “on target” nor “off target” mechanisms. It can alter tumor cell susceptibility to the inhibition of target oncoproteins. Small cell lung cancer (SCLC) transformation is seen in approximately 3% to 14% of NSCLC cases after first line EGFR TKI treatment.^[4,6]^ In such cases, cytotoxic drugs are commonly used according to the histologic findings. However, transformation to squamous cell carcinoma (SQ) is rare, and a standard therapy has not yet been established. We herein report an EGFR T790M mutation positive lung adenocarcinoma that showed histologic transformation to SQ after erlotinib treatment; thus demonstrating the efficacy of osimertinib. The pharmacokinetics of osimertinib were also investigated in this patient. The purpose of this report is to propose a method for the clinical management of T790M mutation positive SQ transformation from EGFR-mutated lung adenocarcinoma through our patient.

## 2. Case

**Figure 1.**
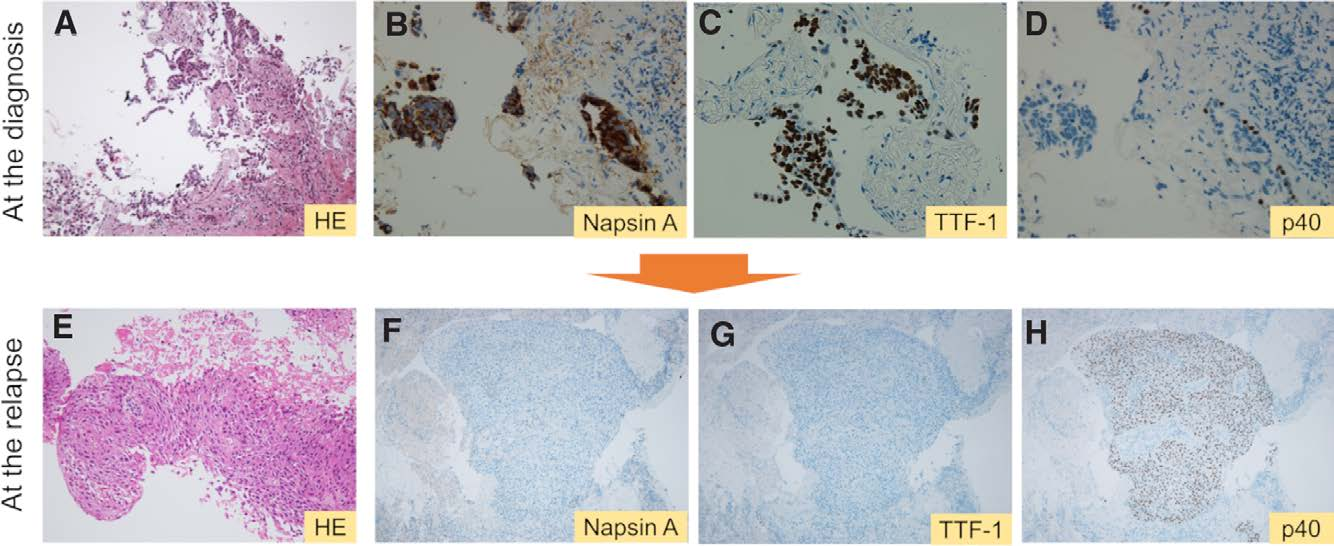
Histology of endobronchial ultrasonography with a guide sheath transbronchial biopsy before erlotinib therapy (A–D). (A) Hematoxylin and eosin (200×), (B) Napsin A (400×), (C) TTF 1 (400×) and (D) p40 staining (400×). Histology of rebiopsy with cryoprobe after erlotinib therapy, suggesting squamous cell transformation (E–H). (E) Hematoxylin and eosin (200×), (F) Napsin A (100×), (G) TTF 1 (100×) and (H) p40 staining (100×).

**Figure 2:**
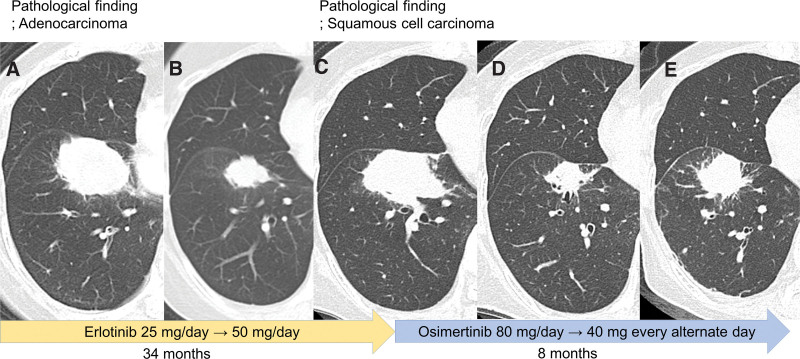
Treatment course with corresponding computed tomography (A–E). (A) before erlotinib treatment, (B) partial response after 5 months erlotinib treatment, (C) disease progression after 34 months erlotinib treatment, (D) partial response after 2 months osimertinib treatment, and (E) disease progression after 8 months of osimertinib treatment.

An 80-year-old nonsmoking woman was referred to our hospital because of an abnormal shadow on her chest radiograph in January 2015 during an annual health check-up. She was asymptomatic and was treated for hypertension and bronchial asthma. Computed tomography (CT) of the chest revealed a nodular shadow in the right lower lung (S8) measuring 26 mm, accompanied by pleural indentation. Diagnostic bronchoscopy was performed using endobronchial ultrasonography with a guide sheath (EBUS-GS) transbronchial biopsy (TBB). Pathological examination revealed adenocarcinoma with napsin A, and the patient tested positive for thyroid transcription factor 1 (TTF-1) and negative for p40 (Fig. 1A–1D). Based on the International Association for the Study of Lung Cancer TNM (8th ed) clinical stages, the patient was diagnosed with stage IB (clinically T2aN0M0) lung cancer. Mutation analysis of the EGFR gene using the cobas® EGFR Mutation Test v2 kit (Roche Diagnostics, USA) revealed deletion of E746-A750 in exon 19. She refused both surgical treatment and radiation therapy, and preferred periodic radiologic follow-up. Unfortunately, approximately a year and a half after the initial diagnosis, the primary lesion enlarged, and many pleural nodules were newly detected (clinically T4N2M1a, stage IVA) (Fig. 2A). The serum carcinoembryonic antigen (CEA) level (1.7 ng/mL) was within the normal limit. From November 2016, based on EGFR mutation analysis, a reduced dose of daily erlotinib was prescribed,^[7]^ which achieved a confirmed partial response (Fig. 2B) and 34 months of progression-free survival (PFS).

In October 2019, repeated biopsy with an endobronchial cryoprobe was performed on the enlarged primary lesion (Fig. 2C). Pathological examination revealed SQ features that were negative for napsin A and TTF 1 and positive for p40, which completely differed from the histological features at initial diagnosis (Fig. 1E–1H). Serum tumor marker did not show elevation of CEA (1.2 ng/mL); however, squamous cell carcinoma related antigen (SCC) (2.0 ng/mL) and cytokeratin 19 fragment (CYFRA) (3.9 ng/mL) were beyond the upper limit of the normal range. Oncomine^TM^ Dx Target Test Multi-CDx system (Thermo Fisher Scientific, USA) revealed that tumor cells harbored an identical EGFR mutation (deletion E746-A750 in exon 19) with a secondary EGFR T790M mutation. According to the EGFR mutation analysis, osimertinib 80 mg once a day was started as second line therapy, which resulted in a partial response in 2 months (Fig. 2D). However, at the same time, osimertinib was discontinued owing to adverse events, grade 3 diarrhea, and grade 2 anorexia. The reduction of osimertinib to 40 mg once daily also caused the same events. After both adverse events resolved during drug holidays, osimertinib was restarted at 40 mg every other day, which resulted in 8 months of PFS and 15 months of survival from the beginning of osimertinib treatment. The enlarged primary lesion after the initiation of osimertinib is shown in Figure 2E. The steady state trough concentration of repeated doses of osimertinib at 40 mg every other day was 446.5 nM (Table 1).^[7,8]^

**Table 1:** The steady state trough concentration of erlotinib and osimertinib.

Treatment	Dose	The steady state trough concentration measured (nM)	The steady state trough concentration previously reported (nM)^[7,8]^
Erlotinib	25 mg	428.4	620.0
Osimertinib	80 mg	N/A	318
	40 mg	N/A	183
	40 mg every other day	446.5	N/A

## 3. Discussion

We encountered a patient who showed transformation from adenocarcinoma to SQ after first line erlotinib treatment and had a significant response to second-line osimertinib treatment. Proper dose reduction of osimertinib in response to adverse events was also proven by measuring the drug concentration.

Although transformation from adenocarcinoma to SQ is uncommon, more cases are being reported.^[9]^ However, its treatment has not yet been standardized. Previous studies have reported poor prognoses in cases that showed transformation from EGFR mutated advanced lung adenocarcinoma to SQ.^[10]^ In our patient, although histologic examination of rebiopsy revealed SQ, panel based next-generation sequencing (NGS) showed EGFR T790M mutation. Eventually, the patient survived for 15 months from the initiation of osimeritinib treatment. This showed the importance of both rebiopsy and treatment selection based on gene profile.

As shown in Table 2,^[10–31]^ a total of 27 cases of SQ transformation in EGFR mutated lung adenocarcinoma after treatment with EGFR TKIs have been reported. The ages of these patients were 40 to 80 years (median age was 62 years), and 18 patients (66.7%) were female. All patients had EGFR mutations, 15 (55.6%) patients had an exon 19 deletion; 10 (37.0%) patients had an L858R point mutation; and 2 (7.4%) patients had other minor mutations. The T790M mutation was detected in only 1 case (3.7%) at the first diagnosis. These characteristics were not significantly different from those of known treatment naïve EGFR mutated NSCLC without SQ transformation in previous reports.^[32,33]^ Moreover, the time from EGFR-TKI initiation to SQ transformation was 4 to 116 months (median duration, 18 months), indicating a trend toward a slightly longer treatment period than that known for EGFR-mutated NSCLC.

**Table 2:** Clinical characteristics of patients with squamous cell carcinoma transformation after resistance to epidermal growth factor receptor tyrosine kinase inhibitors.

No.	Case	Age, sex	Smoking	Original EGFR mutation	Response	TKI, duration before SQT	Chemotherapy before SQT*	Second histology	Duration from TKI initiation to SQT	Acquired gene alterations	Use of osimertinib as subsequent therapy	Survival from SQT
1	Scher et al^[11]^ 2011	58, F	Former	Del19	N/A	Erlotinib 9 m	+	SQ	20 m	–	–	N/A
2	Hsieh et al^[12]^ 2015	51, F	Never	Del19	PR	Gefitinib 4 m	–	SQ	4 m	–	–	>6 m
3	Hsieh et al^[12]^ 2015	61, F	Never	L858R	PR	Gefitinib 12 m	+	SQ	22 m	–	–	N/A
4	Levin et al^[13]^ 2015	66, F	Never	Del19	PR	Erlotinib 8 m	+	SQ	8 m	–	–	N/A
5	Kuiper et al^[14]^ 2015	63, F	Never	L858R	N/A	Erlotinib 5 m	+	SQ	6 m	PIK3CA exon20	–	8 m
6	Jukna et al^[15]^ 2016	74, F	Former	L858R	PR	Gefitinib 10 m	–	SQ	10 m	T790M	–	>11 m
7	Jukna et al^[15]^ 2016	79, F	Never	Del19	PR	Gefitinib 19 m	–	SQ	19 m	T790M	–	>7 m
8	Haratani et al^[16]^ 2016	48, F	Never	Del19	N/A	Gefitinib 24 m	+	SQ	30 m	–	–	N/A
9	Haratani et al^[16]^ 2016	64, F	Never	L858R, T790M	N/A	Gefitinib	+	SQ	N/A	–	–	>10 m
10	Okabe et al^[17]^ 2017	69, M	Former	Del19	PR	Erlotinib 12 m	–	SQ	12 m	T790M	+	>3 m
11	Longo et al^[18]^ 2017	43, F	Former	L858R	PR	Gefitinib 8 m	–	SQ	9 m	S768I	–	2 m
12	Bruno et al^[19]^ 2017	44, F	Former	Del19	PR	Afatinib 18 m	–	SQ	18 m	T790M	+	>2 m
13	Park et al^[20]^ 2017	40, M	Current	Del19	PR	Afatinib 24 m	+	SQ	24 m	T790M	N/A	N/A
14	Izumi et al^[21]^ 2018	68, M	Former	L858R	PR	Erlotinib 9 m	+	SQ	11 m	T790M	+	6 m
15	Kong et al^[22]^ 2018	64, F	Never	L858R	PR	Afatinib 8 m	+	SQ	15 m	T790M	+	>4 m
16	Shinohara et al^[23]^ 2018	62, M	Never	L858R	N/A	Gefitinib 4 m	+	SQ	4 m	–	–	6 m
17	Yao et al^[24]^ 2018	41, M	Current	Del19	PR	Gefitinib 15 m, Osimertinib	+	SCLC → SQ	31 m	N/A	–	1 m
18	Sato et al^[25]^ 2018	52, F	Former	Del19	PR	Erlotinib 12 m	+	SQ	17 m	–	–	>12 m
19	Yamaguchi et al^[26]^ 2019	73, M	Former	Del19	PR	Afatinib 10 m	–	SQ	10 m	T790M	+	>12 m
20	Roca et al^[10]^ 2019	67, F	N/A	L858R → T790M	PR	Gefitinib 58 m, Osimertinib 9 m	–	SQ	67 m	–	–	3 m
21	Chiang et al^[27]^ 2020	54, F	Never	L833V, H835L → T790M	PR	Gefitinib 24 m, Erlotinib 35 m, Afatinib 10 m, Osimertinib 19 m	+	SQ	116 m	mTOR amplification	+	>4 m
22	Uruga et al^[28]^ 2020	61, M	Former	Insertion19	PR	Erlotinib 28 m	+	ASQ → SQ	>42 m	T790M	+	17 m
23	Uruga et al^[28]^ 2020	72, M	Former	L858R	PR	Erlotinib 9 m	–	ASQ	>9 m	T790M	+	8 m
24	Haruki et al^[29]^ 2020	56, F	Never	Del19	PR	Gefitinib 72 m	+	ASQ	72 m	T790M	+	>30 m
25	Hakozaki et al^[30]^ 2020	70, F	Never	Del19	N/A	Gefitinib, Erlotinib	+	SCLC, SQ	N/A	–	–	>4 m
26	Lee et al^[31]^ 2021	44, M	Never	Del19 → T790M	N/A	Gefitinib 10 m, Osimertinib 30 m	+	SQ	45 m	–	+	13 m
27	This case	80, F	Never	Del19	PR	Erlotinib 34 m	–	SQ	34 m	T790M	+	15 m

The positive rate of T790M during the treatment of 27 cases was 59.3%, which was similar to that in a previous report.^[3]^ Osimertinib use after SQ transformation was observed in 40.7% of the 11 patients. The median survival time was estimated to be at least 8 months. In addition, the median survival time was at least 6 months in patients without osimertinib, suggesting that the effect of osimertinib is likely underestimated. These findings suggest that, unlike small cell carcinoma transformation,^[34]^ driver-mutation specific treatment strategies may be beneficial for survival in SQ transformation.

Moreover, we measured the steady state trough concentration of osimertinib when it was prescribed at 40 mg every other day (Table 1). Its concentration was 446.5 nM, which was higher than that which was previously reported (318 nM) at 80 mg every day.^[8]^ In this patient, prolonged high drug concentrations could induce gastrointestinal adverse events such as diarrhea and anorexia. This suggests that dose reduction and individualization are required for osimertinib treatment.

There are some considerable limitations in this report. Firstly, in our patient, we should consider the possibility that the tumor contained an adenosquamous component from the beginning. The possibility of SQ transformation is more likely than the possibility of SQ selection from adenosquamous epithelium because the time from EGFR-TKI initiation to SQ appearance was relatively long and tissue of SQ transformed part detected T790M by NGS. Second, the treatment of SQ transformation is still controversial. The preceding research included patients in whom osimertinib did not always show a response despite the presence of the T790M mutation. Third, publication bias should be taken into account, as there may be published cases where the drug has shown more benefit. Clinical studies are needed to prove the efficacy of osimertinib in patients with SQ transformation and T790M.

## 4. Conclusions

This case report and literature review highlighted the importance of confirming the genetic profile of EGFR mutated lung adenocarcinoma showing SQ transformation as resistance to EGFR TKI, and treatment with individualized EGFR TKI doses. We propose osimertinib is eligible for the treatment of SQ transformation from EGFR-mutated lung adenocarcinoma with T790M mutation.

## Acknowledgment

We would like to thank Editage (www.editage.com) for English language editing.

## Author contributions

Conceptualization: Yusaku Kusaba, Yuichiro Takeda

Data curation: Yusaku Kusaba, Sakurako Abe

Writing - original draft: Yusaku Kusaba, Yuichiro Takeda

Writing - review & editing: Sakurako Abe, Akinari Tsukada, Go Naka.

## Footnotes

* Including 9 cases of platinum plus pemetrexed, 2 cases of tegafur uracil, 2 cases of gemcitabine, and 2 cases of vinorelbine.
